# Discrepancies in ChatGPT’s Hip Fracture Recommendations in Older Adults for 2021 AAOS Evidence-Based Guidelines

**DOI:** 10.3390/jcm13195971

**Published:** 2024-10-08

**Authors:** Hong Jin Kim, Pil Whan Yoon, Jae Youn Yoon, Hyungtae Kim, Young Jin Choi, Sangyoon Park, Jun-Ki Moon

**Affiliations:** 1 Department of Orthopaedic Surgery, Kyung-in Regional Military Manpower Administration, Suwon 16440, Republic of Korea; hongjin0925@naver.com; 2 Department of Orthopedic Surgery, Inje University Sanggye Paik Hospital, Seoul 01757, Republic of Korea; mofi1027@naver.com (H.K.); waiting_4u@nate.com (S.P.); 3 Department of Orthopaedic Surgery, Seoul Now Hospital, Anyang-si 14058, Republic of Korea; orthoyoon@gmail.com (P.W.Y.); dangshei@gmail.com (J.Y.Y.); 4 Department of Orthopedic Surgery, Chung Goo Sung Sim Hospital, Seoul 03330, Republic of Korea; youngjin5771@naver.com; 5 Department of Orthopaedic Surgery, Chung-Ang University Hospital, Seoul 06973, Republic of Korea

**Keywords:** decision-making, hip fracture, AAOS guideline, ChatGPT, artificial intelligence

## Abstract

**Background**: This study aimed to assess the reproducibility and reliability of Chat-Based GPT (ChatGPT)’s responses to 19 statements regarding the management of hip fractures in older adults as adopted by the American Academy of Orthopaedic Surgeons’ (AAOS) evidence-based clinical practice guidelines. **Methods**: Nineteen statements were obtained from the 2021 AAOS evidence-based clinical practice guidelines. After generating questions based on these 19 statements, we set a prompt for both the GPT-4o and GPT-4 models. We repeated this process three times at 24 h intervals for both models, producing outputs A, B, and C. ChatGPT’s performance, the intra-ChatGPT reliability, and the accuracy rates were assessed to evaluate the reproducibility and reliability of the hip fracture-related guidelines. Regarding the strengths of the recommendation compared with the 2021 AAOS guidelines, we observed accuracy of 0.684, 0.579, and 0.632 for outputs A, B, and C, respectively. **Results**: The precision was 0.740, 0.737, and 0.718 in outputs A, B, and C, respectively. For the reliability of the strengths of the recommendation, the Fleiss kappa was 0.409, indicating a moderate level of agreement. No statistical differences in the strengths of the recommendation were observed in outputs A, B, and C between the GPT-4o and GPT-4 versions. **Conclusion**: ChatGPT may be useful in providing guidelines for hip fractures but performs poorly in terms of accuracy and precision. However, hallucinations remain an unresolved limitation associated with using ChatGPT to search for hip fracture guidelines. The effective utilization of ChatGPT as a patient education tool for the management of hip fractures should be addressed in the future.

## 1. Introduction

Large language models (LLMs) in artificial intelligence (AI) have recently been extended to various areas within medicine [1,2]. Since the application of AI technology in the recognition of medical imaging, advances in natural language processing have significantly improved the accessibility for both medical experts and the general population [2]. Based on artificial neural networks, LLMs can generate natural language by understanding statistical patterns within vast amounts of text through a highly computational and supervised training process [3]. With the recent development of Chat-Based GPT (ChatGPT) from Open AI (Open AI, L.L.C.), there has been considerable interest in whether ChatGPT can achieve knowledge levels comparable to humans in the medical field, with studies reporting promising results [4,5,6,7,8,9]. Specifically, LLMs in medicine, as complex network-based transformer models, can comprehensively interpret and understand the nuances of medical language and concepts, allowing them to contextualize information within the broader scope of patient care [1,2,3]. Beyond understanding statistical patterns, they can synthesize data from diverse sources to generate coherent and relevant responses to complex clinical queries [1].

Many patients, as well as healthcare users, easily search for and rely on individual medical information using web-based sources, which serve as helpful tools [10,11]. Growing in popularity, ChatGPT’s responses to common patient questions regarding diseases have been studied and found to provide sufficiently informative answers [5,8,9]. Currently, these models function as auxiliary means rather than decision-making tools, but there is a lack of research on whether they can clearly fulfill this role [3]. In the field of orthopedic surgery, an assessment of ChatGPT’s responses also revealed that the chatbot provided evidence-based responses to questions commonly asked by patients regarding total hip arthroplasty, hip arthroscopy, and patient-reported outcome measures [5,8,12]. Specifically, regarding hip fractures, Wrenn et al. reported that ChatGPT provided unbiased and evidence-based answers that were clearly understood by most orthopedic patients [9].

However, from an orthopedic surgeon’s perspective, unsolved and conflicting issues remain in the decision-making process for the management of hip fractures in older adults, such as preoperative traction, cemented femoral stems, and surgical approaches [13,14]. Specifically, the evidence-based clinical practice guidelines adopted by the American Academy of Orthopaedic Surgeons’ (AAOS) Board of Directors have been presented as the current best evidence associated with treatment, which is intended for use by orthopedic surgeons and other healthcare providers [15]. It is unknown whether ChatGPT can respond sufficiently to these specialized areas regarding the management of hip fractures in older adults. Therefore, this study aimed to assess the reproducibility and reliability of ChatGPT’s responses to 19 statements regarding the management of hip fractures in older adults as adopted by the AAOS evidence-based clinical practice guidelines.

## 2. Materials and Methods

### 2.1. 2021 AAOS Guidelines for Management of Hip Fractures in Older Adults

A total of 19 statements related to the management of hip fractures in older adults were obtained from the evidence-based clinical practice guideline adopted by the AAOS’ Board of Directors (3 December 2021) [15]. For this guideline, there were 16 recommendations and three options for the management of hip fractures in older adults. Recommendations were formulated when sufficient evidence was available to substantiate the directional statements. Evidence regarding the recommendations was determined by two or more quality studies (two or more high-quality studies: strong recommendations; or two or more moderate-quality studies: moderate recommendations) following adjustments based on the Evidence of the Decision Framework. Options were formulated when the topic had little or no evidence in the literature. The evidence regarding recommendations was determined by low-quality evidence or a single moderate-quality study (a limited-strength option), no evidence, or conflicting evidence (a consensus option), following adjustments based on the Evidence of the Decision Framework.

### 2.2. Data Extraction and Generation

**Figure 1:**
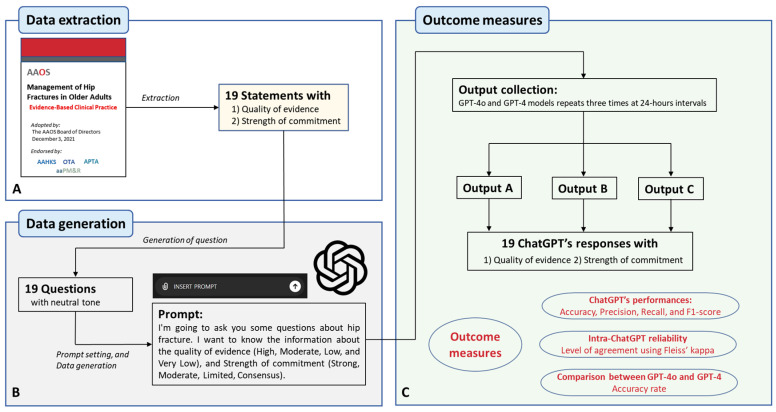
The study flowchart. (**A**) Data extraction protocol, (**B**) data generation using the ChatGPT protocol, and (**C**) outcome measures protocol.

We extracted 19 statements with high-quality evidence and strength of commitment regarding the management of hip fractures in older adults (Figure 1A). These statements, adopted from the 2021 AAOS guidelines, are summarized in Table 1. To generate questions based on these 19 statements, we formulated them in a neutral tone to minimize bias raised by the prompt itself. These questions were then used as prompts for both the GPT-4o and GPT-4 models, respectively: “I’m going to ask you some questions about hip fractures. I want to know information about the quality of evidence (High, Moderate, Low, and Very Low) and the strength of commitment (Strong, Moderate, Limited, Consensus) for our 19 questions” (Figure 1B).

### 2.3. Outcome Measures

We repeated this process three times at 24 h intervals for both the GPT-4o and GPT-4 models, yielding outputs A, B, and C. For the 19 ChatGPT responses at outputs A, B, and C, we collected quality evidence and strengths of commitment. Based on these data, we measured ChatGPT’s performance (including accuracy, precision, recall, and F1-score) and the intra-ChatGPT reliability (level of agreement for outputs A, B, and C) and compared the accuracy rates between the GPT-4o and GPT-4 models (Figure 1C). Regarding the performance of ChatGPT, the accuracy was measured as the ratio of correctly predicted instances to the total number of instances. The precision was defined as the ratio of correctly positive observations to the total number of predicted positives. Recall, also known as sensitivity, was defined as the ratio of correctly predicted positive observations compared to all observations in an actual class. The F1-score was defined as the harmonic mean of the precision and recall.

### 2.4. Statistical Analysis and Visualization

Statistical analyses and visualizations were performed using Python (version 3.11.5., Python Software Foundation, Wilmington, DE, USA) with Matplotlib (version 3.7.2). The normal distribution was confirmed using the Kolmogorov–Smirnov test. After confirming the data homogeneity or heteroscedasticity, Student’s *t*-test was used for continuous variables, and the chi-square test was used for categorical variables, as appropriate. The performance, accuracy, precision, recall, and F1-score were calculated as (true positives [TP] + true negatives [TN])/(TP + TN + false positives [FP] + false negatives [FN]), TP/(TP + FP), TP/(TP + FN), and (2 × precision × recall)/(Precision + Recall), respectively [16,17]. Fleiss’ kappa was calculated to assess the level of agreement among the reliability of the three ChatGPT responses (outputs A, B, and C). Statistical significance was set at a two-tailed *p* < 0.05.

## 3. Results

### 3.1. Performance and Reliability of ChatGPT-Generated Responses (GPT-4o Version)

The answers (quality of evidence and strength of recommendation in outputs A, B, and C) to the 19 questions regarding the 2021 AAOS guidelines for the management of hip fractures in older adults are described in Table 2. In these data, ChatGPT’s responses to the same prompts could differ from one another (outputs A, B, and C). For instance, the quality of evidence was suggested as high in outputs A and B but moderate in output C for the preoperative content. The strength of recommendation was indicated as limited in outputs A and B but as consensus in output C for the surgical approach.

With regard to the quality of evidence compared to the results of the 2021 AAOS guidelines, the accuracy was 0.684 for output A, 0.579 for output B, and 0.579 for output C. The precision was 0.585 in output A, 0.675 in output B, and 0.522 in output C. The recall was the same as the accuracy. The F1-scores were 0.629, 0.581, and 0.549 for outputs A, B, and C, respectively. With regard to the evaluation of the reliability between outputs A, B, and C, the Fleiss kappa was 0.266 (95% CI: 0.248–0.340, *p* < 0.001), indicating a fair level of agreement (Table 3).

With regard to the strength of the recommendation compared with the results of the 2021 AAOS guidelines, the accuracy was 0.684 for output A, 0.579 for output B, and 0.632 for output C. The precision was 0.740 in output A, 0.737 in output B, and 0.718 in output C, respectively. The recall was the same as the accuracy. The F1-scores were 0.630, 0.624, and 0.597 for outputs A, B, and C, respectively. To evaluate the reliability between outputs A, B, and C, the Fleiss kappa was 0.409 (95% CI: 0.119–0.699, *p* = 0.006), indicating a moderate level of agreement (Table 4).

### 3.2. Comparison of ChatGPT’s Responses between the GPT-4o and GPT-4 Versions

**Figure 2:**
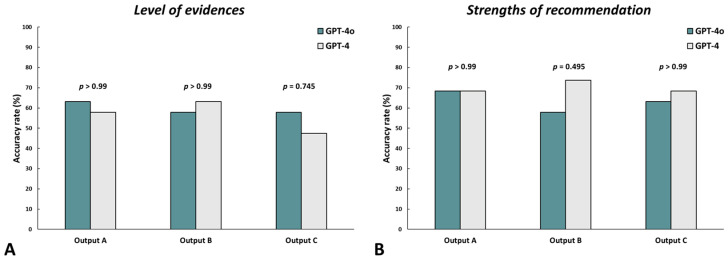
Comparison of accuracy rate between GPT-4o and GPT-4 versions. (**A**) Level of evidence and (**B**) strength of recommendation.

We also assessed the accuracy rate to compare the ChatGPT responses between the GPT-4o and GPT-4 versions. With regard to the level of evidence, the accuracy rate exhibited no statistical differences in outputs A (*p* > 0.99), B (*p* > 0.99), and C (*p* = 0.745) between the GPT-4o and GPT-4 versions (Figure 2A). With regard to the strengths of the recommendations, no statistical differences were observed in outputs A (*p* > 0.99), B (*p* = 0.495), and C (*p* > 0.99) between the GPT-4o and GPT-4 versions (Figure 2B).

## 4. Discussion

ChatGPT, as an innovative development of AI technology, has grown in popularity since its public release [1]. The use of ChatGPT in medicine is advantageous owing to its accessibility to patients as a search engine, providing information on various diseases or conditions, including hip fractures [1,6,9]. Hip fractures are significant healthcare concerns, particularly in older populations, affecting 18% of women and 6% of men globally [13,18]. As one of the leading causes of hospitalization in older groups, hip fractures do not only pose socioeconomic burdens but are also projected to rise to 4.5 million cases by 2050 [18,19]. Therefore, the management of hip fractures is currently a major focus in geriatric medicine for both healthcare specialists and patients [13]. Although ChatGPT can be considered as a patient education tool regarding proper responses for common orthopedic injuries, it remains unknown whether ChatGPT can accurately respond to specific questions regarding the recommended guidelines for the management of hip fractures [6,20]. The main goal of this study was to assess the reproducibility and reliability of ChatGPT’s responses to a total of 19 statements related to the management of hip fractures in older adults as adopted by the AAOS evidence-based clinical practice guidelines. From this study, we found that the accuracy rate of the “recommendation” ranged from 57.9 to 68.4%, and the reliability was found to be at a moderate level of agreement. Furthermore, there were no statistical differences in the accuracy rates according to the ChatGPT version.

With the technical development of LMMs, several studies have documented ChatGPT’s responses to common questions related to orthopedic diseases [5,8,9,12]. AlShehri et al. demonstrated that ChatGPT can sufficiently answer common patient questions regarding hip arthroscopy, as graded by two hip surgeons [5]. However, incorrect information was also identified, necessitating caution in patient education [5]. Meanwhile, Mika et al. demonstrated that ChatGPT’s responses to questions regarding hip arthroplasty provided evidence-based information, serving as a valuable clinical tool for patient education prior to orthopedic consultations [8]. Furthermore, Wrenn et al. suggested that ChatGPT could provide unbiased, evidence-based answers to frequently asked questions about hip fractures [9]. Despite several survey studies for commonly asked questions in hip diseases, it is not yet known whether ChatGPT can provide appropriate answers for specialized areas that remain controversial [7]. Our research focuses on these specialized and conflicting aspects of hip fractures and the capability of ChatGPT to contrast with responses to commonly asked questions. The accuracy rate of up to 68.4% suggests that ChatGPT may still be associated with errors when it comes to providing orthopedic guidelines for the management of hip fractures in older adults. Furthermore, our findings indicate that ChatGPT may provide different answers to the same prompt, with accuracy ranging from 0.579 to 0.684 regarding the quality of evidence and strength of recommendations in our study. Considering its use as a supportive tool, it is important to take a careful approach due to the potential to suggest misinformation.

AI hallucination is a significant problem as it can provide incorrect or misleading information to patients [21,22,23]. It is defined as the phenomenon whereby LMMs generate incorrect or non-sensical text regardless of the use of pre-trained data [22,23]. Because high accuracy is essential to patient education in healthcare, particular caution regarding this phenomenon and efforts to identify AI hallucinations are necessary when assessing ChatGPT’s responses to commonly asked questions regarding hip fractures. Precision indicates the proportion of correctly generated information out of all information provided by the model, which is mainly associated with hallucinations [23]. In other words, a high precision rate is necessary to reduce hallucinations. Our study indicated that the precision rate for the strengths of the recommendations ranged from 71.8 to 74.0%, suggesting an insufficient level of healthcare education in the management of hip fractures. Furthermore, our findings indicate that hallucinations remain an unsolved issue in the application of LLMs and must be addressed before ChatGPT can be effectively utilized for patient education in the future. Various efforts in data quality, pre-training, fine-tuning, feedback, and iterative learning are essential to reduce hallucinations. The ChatGPT model itself has evolved and developed through enhanced training data and model architectures, reinforcement learning from human feedback, and acknowledging uncertainty. Therefore, professional participation in providing human feedback can help to reduce confabulations, with the potential to establish the model as a supportive educational tool in the field of orthopedic surgery.

The role of AI has evolved to enhance diagnostic accuracy, personalize treatment plans, and improve surgical outcomes. In orthopedic surgery, the advancement of AI has contributed to its supportive role in preoperative decision-making. Meanwhile, LLMs in medicine can serve as a supportive option as a patient and surgeon educational tool. Despite this potential, our research results suggest that the performance of LLM models is still insufficient from the perspective of providing a unified service and needs to be further developed. To minimize the confabulation phenomenon in LLMs, ChatGPT has been developed with a more advanced model. The GPT-4o model is a more advanced version of the ChatGPT GPT-4 version, featuring improvements in both performance and efficiency [24]. The GPT-4o model enhances natural language processing (better handling of context), reduces the hallucination rate, and generates more reliable information [24]. Although the advanced version of ChatGPT (GPT-4o) enables faster responses and has lower computational costs, no statistical difference in the accuracy rate was observed between the two versions [24]. However, it should be noted that ChatGPT does not yet provide sufficiently reliable responses for the management of hip fracture-related recommendations when we consider hallucinations. Therefore, further development to resolve hallucinations is essential in providing a supportive role as a patient education tool.

Nietsch et al. recently reported on the ability of ChatGPT-4.0 to predict appropriate treatments for acute hip fractures in older adults [25]. In their study, they created 30 patient scenarios for 180 paired scores, which were divided among six treatment options: total hip arthroplasty, hemiarthroplasty, long cephalomedullary nails, short cephalomedullary nails, sliding hip screws, and multiple cannulated screws. They found that ChatGPT-4.0 provided results comparable to the AAOS Appropriate Use Criteria for five of the treatment options, with the exception of long cephalomedullary nails. In contrast, our study evaluated ChatGPT’s responses to each of the 19 statements presented by the 2021 AAOS guidelines in a comprehensive manner. Using a study design distinct from the aforementioned study, we demonstrated that ChatGPT is currently insufficient in providing specialized and appropriate information regarding the surgical treatment of acute hip fractures [25]. We believe that this limitation is largely due to hallucinations, which remain a significant issue for LLMs [21,22,23]. Furthermore, we compared the reliability of the GPT-4 version with that of the newer, more advanced GPT-4o version. While the upgrade to GPT-4o reduced AI hallucinations, it is still insufficient in providing as much detailed information as the 2021 AAOS guidelines, based on our findings [24]. Therefore, both surgeons and patients should be cautious when seeking information from LLM models, as their performance may not yet be sufficient to achieve appropriate outcomes.

The clinical application of LLMs is controversial. In particular, the deficiencies of AI in medicine have been addressed in areas from educational tools to clinical decision-making [26,27]. Since the nature of AI tools has misconceptions and biases, newer clinical information, suggested by high-quality studies, is challenging. This is thought to be the result of ChatGPT’s mechanism, stemming from the process of elaborating after data crawling, which is inevitably different from actual intelligence [27]. In our study, several of ChatGPT’s answers were not entirely precise, which is commonly found in research on the reliability of medical information using LLMs [26,27]. Therefore, to develop appropriate clinical support tools for decision-making and education, it is necessary for healthcare professionals to perform quality control and the critical assessment of the outputs generated from prompts.

This study had several limitations. First, a standardized assessment tool for ChatGPT does not currently exist; therefore, inaccurate analyses remain an unresolved issue. Even so, we tried to compare the reproducibility based on objectified data (such as “quality of evidence” and “strengths of recommendations”) as much as possible. We used classical performance measures including accuracy, precision, recall, and the F1-score. However, these measures have limitations that can affect their ability to fully represent a model’s performance. Accuracy is not only sensitive to class imbalance but also does not differentiate between error types, such as false positives and false negatives, which can potentially give a misleading impression of the model’s true performance. Meanwhile, the F1-score, despite being the harmonic mean of the precision and recall, does not reflect true negative values. Furthermore, this score assumes that precision and recall are equally weighted, so relying solely on the F1-score can be misleading. In other words, the research methodology we adopted has limitations. Therefore, a comprehensive approach using complementary metrics, such as the area under the receiver operating characteristic curve, will be required in the future. However, it is necessary to establish a clear research methodology for the clinical application of LLMs. Nevertheless, our study explored whether it could be used as an appropriate auxiliary means by investigating the performance regarding the 2021 AAOS guidelines on hip fractures. Second, ChatGPT can elicit various responses according to different prompts. However, our study was conducted using a single prompt to minimize the variation in the output caused by prompts. Therefore, a highly sensitive output based on prompts is a characteristic of ChatGPT, which should be addressed regarding the responses to commonly asked questions about hip fractures. Third, ChatGPT is evolving and can be developed using new data and user feedback at regular intervals, potentially enhancing its performance and accuracy in the future.

## 5. Conclusions

ChatGPT may be useful in searching for guidelines for the management of hip fractures, but, as a patient educational tool, it performs poorly in terms of accuracy and precision, partly due to AI hallucinations. Therefore, AI hallucinations are currently an unresolved limitation associated with the use of ChatGPT to search for hip fracture management guidelines. The utilization of ChatGPT as a patient educational tool for nuanced medical advice regarding the management of hip fractures must be addressed in future studies.

## Figures and Tables

**Table 1:** The 2021 AAOS guidelines for the management of hip fractures in older adults.

Content	Guideline	QE	SR
Preoperative traction	Preoperative traction should not routinely be used for patients with a hip fracture.	High	Strong
Surgical timing	Hip fracture surgery within 24–48 h of admission may be associated with better outcomes.	Low	Moderate
VTE prophylaxis	VTE prophylaxis should be used in hip fracture patients.	Moderate	Strong
Anesthesia	Either spinal or general anesthesia is appropriate for patients with a hip fracture.	High	Strong
Unstable femoral neck fractures: arthroplasty vs. fixation	In patients with unstable (displaced) femoral neck fractures, arthroplasty is recommended over fixation.	High	Strong
Unipolar/bipolar hemiarthroplasty	In patients with unstable (displaced) femoral neck fractures, unipolar or bipolar hemiarthroplasty can be equally beneficial.	Moderate	Moderate
Unstable femoral neck fractures: total arthroplasty vs. hemiarthroplasty	In properly selected patients with unstable (displaced) femoral neck fractures, there may be a functional benefit to total hip arthroplasty over hemiarthroplasty at the risk of increasing complications.	High	Moderate
Cemented femoral stems	In patients undergoing arthroplasty for femoral neck fractures, the use of cemented femoral stems is recommended.	High	Strong
Surgical approach	In patients undergoing treatment for femoral neck fractures with hip arthroplasty, evidence does not show a favored surgical approach.	Moderate	Moderate
Cephalomedullary device: stable intertrochanteric fractures	In patients with stable intertrochanteric fractures, the use of either a sliding hip screw or a cephalomedullary device is recommended.	High	Strong
Cephalomedullary device: subtrochanteric/reverse obliquity fractures	In patients with subtrochanteric or reverse obliquity fractures, a cephalomedullary device is recommended.	High	Strong
Cephalomedullary device: unstable intertrochanteric fractures	Patients with unstable intertrochanteric fractures should be treated with a cephalomedullary device.	High	Strong
Transfusion	A blood transfusion threshold of no higher than 8 g/dL is suggested in asymptomatic postoperative hip fracture patients.	Moderate	Moderate
Multimodal analgesia	Multimodal analgesia incorporating a preoperative nerve block is recommended to treat pain after hip fractures.	High	Strong
TXA	TXA should be administered to reduce blood loss and blood transfusion in patients with hip fractures.	High	Strong
Interdisciplinary care programs	Interdisciplinary care programs should be used in the care of hip fracture patients to decrease complications and improve outcomes.	High	Strong
Stable femoral neck factures	In patients with stable (impacted/non-displaced) femoral neck fractures, hemiarthroplasty, internal fixation, or non-operative care may be considered.	Moderate	Limited
Cephalomedullary device: pertrochanteric fractures	In patients with pertrochanteric femur fractures, a short or long cephalomedullary nail may be considered.	Low	Limited
Weight bearing	Following the surgical treatment of hip fractures, immediate, full weight bearing to tolerance may be considered.	Low	Limited

Quality of evidence: high, moderate, low, and very low; strengths of recommendation or option: strong, moderate, limited, and consensus. QE, quality of evidence; SR, strength of recommendation; VTE, venous thromboembolism prophylaxis; TXA, tranexamic acid.

**Table 2:** ChatGPT’s responses (GPT-4o version) regarding the 2021 AAOS guidelines for the management of hip fractures in older adults.

Content	Questions for the Prompt	Output A		Output B		Output C	
		QE	SR	QE	SR	QE	SR
Preoperative traction	Is preoperative traction not routinely used for patients with a hip fracture?	High	Strong	High	Strong	Moderate	Strong
Surgical timing	Is hip fracture surgery within 24–48 h of admission associated with better outcomes?	High	Strong	Moderate	Strong	Moderate	Strong
VTE prophylaxis	Is venous thromboembolism (VTE) prophylaxis used in hip fracture patients?	High	Strong	Moderate	Strong	High	Strong
Anesthesia	Is either spinal or general anesthesia appropriate for patients with a hip fracture?	Moderate	Strong	Moderate	Consensus	High	Strong
Unstable femoral neck fractures: arthroplasty vs. fixation	In patients with unstable (displaced) femoral neck fractures, is arthroplasty recommended over fixation?	High	Strong	High	Strong	High	Strong
Unipolar/bipolar hemiarthroplasty	In patients with unstable (displaced) femoral neck fractures, are unipolar and bipolar hemiarthroplasty equally beneficial?	Moderate	Limited	Moderate	Consensus	Moderate	Limited
Unstable femoral neck fractures: total arthroplasty vs. hemiarthroplasty	In properly selected patients with unstable (displaced) femoral neck fractures, is there a functional benefit to total hip arthroplasty over hemiarthroplasty despite the risk of increasing complications?	High	Strong	Moderate	Moderate	Moderate	Moderate
Cemented femoral stems	In patients undergoing arthroplasty for femoral neck fractures, is the use of cemented femoral stems recommended?	Moderate	Strong	High	Strong	High	Strong
Surgical approach	In patients undergoing treatment of femoral neck fractures with hip arthroplasty, does evidence show a favored surgical approach?	Moderate	Limited	Low	Limited	Low	Consensus
Cephalomedullary device: stable intertrochanteric fractures	In patients with stable intertrochanteric fractures, is the use of either a sliding hip screw or a cephalomedullary device recommended?	High	Strong	Moderate	Consensus	High	Strong
Cephalomedullary device: subtrochanteric/reverse obliquity fractures	In patients with subtrochanteric or reverse obliquity fractures, is a cephalomedullary device recommended?	High	Strong	Moderate	Strong	High	Strong
Cephalomedullary device: unstable intertrochanteric fractures	In patients with unstable intertrochanteric fractures, is a cephalomedullary device used?	High	Strong	High	Strong	High	Strong
Transfusion	Is a blood transfusion threshold of no higher than 8 g/dL suggested in asymptomatic postoperative hip fracture patients?	Moderate	Moderate	Moderate	Moderate	Moderate	Strong
Multimodal analgesia	Is multimodal analgesia incorporating preoperative nerve block recommended to treat pain after a hip fracture?	High	Strong	High	Strong	High	Strong
TXA	Is TXA administered to reduce blood loss and blood transfusion in patients with hip fractures?	High	Strong	High	Strong	High	Strong
Interdisciplinary care programs	Are interdisciplinary care programs used in the care of hip fracture patients to decrease complications and improve outcomes?	High	Strong	High	Strong	High	Strong
Stable femoral neck factures	In patients with stable (impacted/non-displaced) femoral neck fractures, are hemiarthroplasty, internal fixation, or non-operative care considered?	Moderate	Strong	Moderate	Consensus	High	Consensus
Cephalomedullary device: pertrochanteric fractures	In patients with pertrochanteric femur fractures, is a short or long cephalomedullary nail considered?	Moderate	Limited	Moderate	Consensus	Moderate	Consensus
Weight bearing	Following surgical treatment of hip fractures, is immediate, full weight bearing to tolerance considered?	High	Strong	Moderate	Strong	High	Strong

Quality of evidence: high, moderate, low, and very low; strength of recommendation or option: strong, moderate, limited, and consensus. QE, quality of evidence; SR, strength of recommendation; VTE, venous thromboembolism prophylaxis; TXA, tranexamic acid.

**Table 3:** ChatGPT’s performance and reliability for the “quality of evidence” in the 2021 AAOS guidelines for the management of hip fractures in older adults.

**ChatGPT’s Performance (GPT-4o Version)**				
**Model**	Accuracy	Precision (=PPV)	Recall (=Sn)	F1-Score
Output A	0.684	0.585	0.684	0.629
Output B	0.579	0.675	0.579	0.581
Output C	0.579	0.522	0.579	0.549
**Reliability Test (GPT-4o Version)**				
**Variables**	Fleiss’ Kappa	Level of Agreement	95% CI	*p*
Reliability	0.266	Fair	0.248–0.340	<0.001

The quality of evidence was divided into high, moderate, low, and very low and was validated based on whether the 2021 AAOS guidelines matched each output A, B, and C. PPV, positive predictive value; Sn, sensitivity.

**Table 4:** ChatGPT’s performance and reliability for the “strength of recommendation” in the 2021 AAOS guidelines for the management of hip fractures in older adults.

**ChatGPT’s Performance (GPT-4o Version)**				
**Model**	Accuracy	Precision (=PPV)	Recall (=Sn)	F1-Score
Output A	0.684	0.740	0.684	0.630
Output B	0.579	0.737	0.579	0.624
Output C	0.632	0.718	0.632	0.597
**Reliability test (GPT-4o Version)**				
**Variables**	Fleiss’ Kappa	Level of Agreement	95% CI	*p*
Reliability	0.409	Moderate	0.119–0.699	0.006

The strengths of recommendation were divided into strong, moderate, limited, and consensus and were validated based on whether the 2021 AAOS guidelines matched each output A, B, and C. PPV, positive predictive value; Sn, sensitivity.

## Data Availability

The data presented in this study are available on request from the corresponding author due to privacy restrictions.
